# Assessment of subjective image quality, contrast to noise ratio and modulation transfer function in the middle ear using a novel full body cone beam computed tomography device

**DOI:** 10.1186/s12880-023-00996-6

**Published:** 2023-04-10

**Authors:** Anssi-Kalle Heikkinen, Valtteri Rissanen, Antti A. Aarnisalo, Kristofer Nyman, Saku T. Sinkkonen, Juha Koivisto

**Affiliations:** 1 Department of Otorhinolaryngology-Head and Neck Surgery and Tauno Palva Laboratory, Head and Neck Center, University of Helsinki and Helsinki University Hospital, Helsinki, Finland; 2 Radiology, HUS Diagnostic Center, University of Helsinki and Helsinki University Hospital, Helsinki, Finland; 3 Department of Physics, University of Helsinki, Helsinki, Finland

**Keywords:** Middle ear imaging, Full body CBCT, Cone beam computed tomography, 3D reconstruction, Contrast to noise ratio, CNR, Subjective image quality

## Abstract

**Background:**

Multi slice computed tomography (MSCT) is the most common used method in middle ear imaging. However, MSCT lacks the ability to distinguish the ossicular chain microstructures in detail resulting in poorer diagnostic outcomes. Novel cone beam computed tomography (CBCT) devices’ image resolution is, on the other hand, better than MSCT resolution. The aim of this study was to optimize imaging parameters of a novel full body CBCT device to obtain optimal contrast to noise ratio (CNR) with low effective dose, and to optimize its clinical usability.

**Methods:**

Imaging of five anonymous excised human cadaver temporal bones, the acquisition of the effective doses and the CNR measurements were performed for images acquired on using Planmed XFI® full body CBCT device (Planmed Oy, Helsinki, Finland) with a voxel size of 75 µm. All images acquired from the specimens using 10 different imaging protocols varying from their tube current exposure time product (mAs) and tube voltage (kVp) were analyzed for eight anatomical landmarks and evaluated by three evaluators.

**Results:**

With the exception of protocol with 90 kVp 100 mAs, all other protocols used are competent to image the finest structures. With a moderate effective dose (86.5 µSv), protocol with 90 kV 450 mAs was chosen the best protocol used in this study. A significant correlation between CNR and clinical image quality of the protocols was observed in linear regression model. Using the optimized imaging parameters, we were able to distinguish even the most delicate middle ear structures in 2D images and produce accurate 3D reconstructions.

**Conclusions:**

In this ex vivo experiment, the new Planmed XFI® full body CBCT device produced excellent 2D resolution and easily created 3D reconstructions in middle ear imaging with moderate effective doses. This device would be suitable for middle ear diagnostics and for e.g., preoperative planning. Furthermore, the results of this study can be used to optimize the effective dose by selecting appropriate exposure parameters depending on the diagnostic task.

## Background

Currently, multi slice computed tomography (MSCT) is the most common method in clinical middle ear imaging. It is a valuable and important diagnostic tool for pathologies resulting in conductive hearing loss and in otosurgical planning. Although the MSCT technology has advanced during recent years, it doesn’t always provide accurate enough information of middle ear structures because of the limited slice thickness for distinguishing of microstructures in ossicular chain, especially stapes, and resulting in poorer diagnostic outcomes. In addition, radiation exposure of current MSCT devices are quite high [1].

Cone beam computed tomography (CBCT) imaging technology differs from MSCT. Even though both scanners with their X-ray tubes and detectors rotating cylindrically may look similar, the type of reconstructed images differs. In CBCT, the entire volume of data is captured in a single rotation of cone-shaped continuous or pulsed X-ray beam [2, 3] which, on the other hand, induces higher scatter radiation than that on MSCT devices [4]. In terms of MSCT, the volume is reconstructed by superimposition of slices [1]. CBCT detector is plane-shaped compared to a curved MSCT detector [1]. CBCT voxels are cube-shaped whereas MSCT voxels rarely are and, also, CBCT’s isotropic volume differs from MSCT’s anisotropic volume [1]. In addition, comparing to MSCT, performing a CBCT scan takes shorter time [2] and the effective dose (ED) is lower [5] which can make it more suitable also for pediatric use [1]. Studies show the EDs of CBCT to be 1/5–1/3 compared to MSCT [1, 6–9]. CBCT devices offer better contrast resolution [10] for bone imaging where excellent contrast between bone and mucosa as well as bone and air is needed [1]. Furthermore, CBCT provides more reliable spatial data with higher resolutions when compared to MSCT technology [1].

CBCT also offers better image quality with fewer artifacts for imaging dense metallic structures [11] when compared to MSCT. However, CBCT devices exhibit more beam hardening artifacts [12] and have lower soft tissue contrast discrimination capability than MSCT or magnetic resonance imaging (MRI) techniques [1, 2, 13]. In addition, CBCT images contain more noise resulting from the lower radiation intensity and smaller voxel size when compared to the MSCT scanners [1].

The first CBCT prototypes for middle ear imaging were developed in 2000s although the technology was introduced in 1990s [11, 14]. Previously, CBCT technology has had many applications in maxillofacial and dental surgery but more recently it has suggested to be useful in otology as well [15]. Earlier studies have shown CBCT image quality to be comparable to that of MSCT devices [9, 15, 16]. CBCT technology is currently advancing rapidly and in a recent study the image quality of CBCT was discussed to be superior to MSCT and was considered as a promising alternative to MSCT in clinical use [6].

By optimizing the voltage (kVp) and current exposure time product (mAs), radiological diagnostic objectives can be achieved with fine diagnostic quality [1, 17]. Especially, when imaging middle and inner ear where low signal with small voxel size and fine slice thickness are combined with high spatial resolution, CBCT device exposure parameters need optimization because decreasing voxel size results in an increase of noise and consequently a decrease of signal to noise ratio (SNR) [1]. Image noise can be reduced by increasing the mAs. This, however, increases the ED proportionally. The impact of kVp in image quality is more complex than that of mAs due to the interactions of X-ray photons with the tissues [17]. CBCT imaging parameters used for imaging of excised temporal bones have been introduced in previous studies. For example, in studies by Dietz et al. [18, 19] and Iso-Mustajärvi et al. [20], CBCT imaging parameters were 80–96 kVp, 106.5– 240 mAs and 82– 98 µSv for ED.

The complex bony structure of the temporal bone and aerated middle ear with ossicular chain inside makes it ideal target for CBCT [11] but also challenging environment due to the contrast resolution limitations caused by the image noise [1]. When imaging the middle ear, the soft tissue resolution is limited due to the attenuation caused by the dense bony structures of the temporal bone [21]. A previous study by Zou et al. [22] identified and measured fine human cadaver temporal bone structures with a high-resolution CBCT device with constant imaging parameters and found the utility of the system appropriate in middle ear imaging. Using human cadaver temporal bones, too, a study by Kemp et al. [6] compared quality and EDs of high-resolution CBCT and MSCT in middle ear imaging by evaluating anatomical landmarks of the middle and inner ear. To our knowledge, no published study compares the exposure parameters with the technical and subjective image quality and the ED. The aims of the present study were to assess the impact of imaging parameters of the novel full body CBCT device in middle ear imaging to obtain optimal contrast to noise ratio (CNR) with low ED, and to assess the clinical usability of the device.

## Methods

This human cadaver temporal bone study was conducted to optimize the kVp and mAs with standardized patient ED to achieve the best subjective image quality of eight anatomical landmarks within the middle ear and CNR using a novel full body CBCT (Planmed XFI®, Planmed Oy, Helsinki, Finland). Furthermore, the impact of low ED (low mAs) and high ED using different kVp values to image qualities were investigated.

The study fulfilled the Helsinki Declaration for ethical use of human material. Institutional Review Board (IRB) at Helsinki University Hospital approved the study protocol and the use of anonymous cadaveric temporal bones in the study (Approval No. §49/29.10.2020, HUS/58/2020). The temporal bones were dissected at in the Finnish Institute for Health and Welfare’s Department of Forensic Medicine, Helsinki, with the permission of National Supervisory Authority for Welfare and Health (Permission No. 6834/06.01.03.01/2013). The temporal bones were dissected in a standard way resulting in a specimen with an average size of 8 cm × 6 cm × 5 cm containing all the relevant structures of the middle ear, inner ear, and mastoid air cell system.

### Scanner

**Fig. 1:**
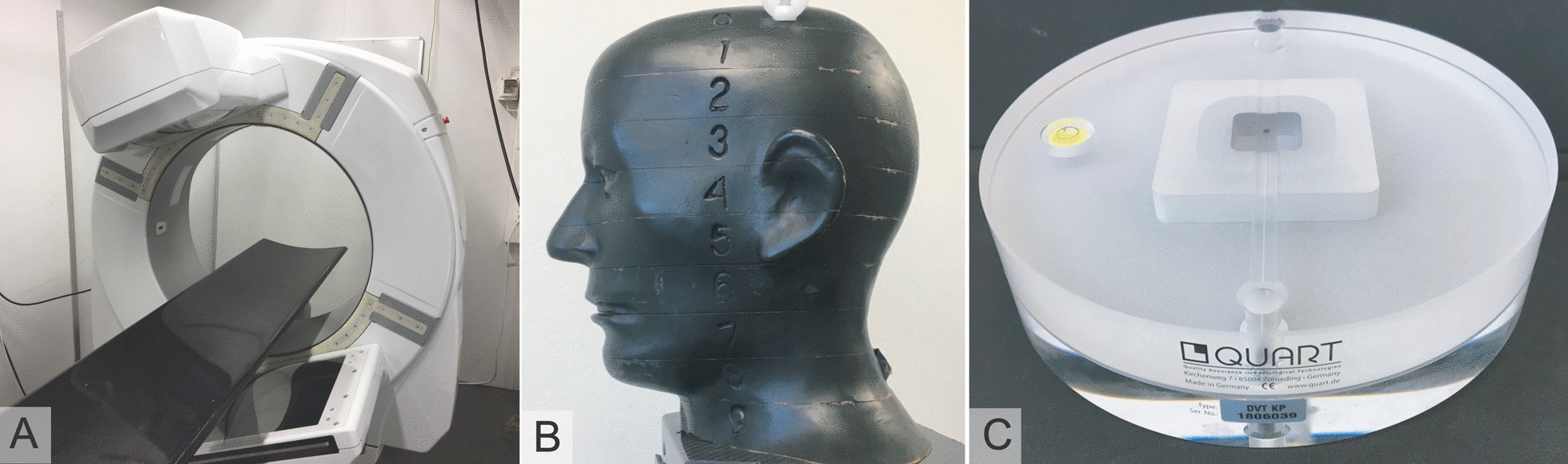
**A** Planmed XFI® full body CBCT device. **B** Anthropomorphic s RANDO RAN 102 phantom. **C.** QUART DVT_AP phantom

Imaging of the cadaver temporal bones, the acquisition of the EDs and the measurements were performed for images acquired on using Planmed XFI® full body CBCT device (Fig. 1A). The scanner has a IAE RTM 782 HS X-ray tube (IAE S.p.A, Cormano, Milano, Italy) with 0.7 mm inherent filtration and 2.5 mm Al + 0.5 mm Cu or 0.2 mm Cu + bowtie added filtrations. The novel full body CBCT scanner of the current study is dedicated for imaging of the head, the extremities, and the torso. The kVp range can be adjusted between 80–140 kVp, the tube mAs can be adjusted depending on kVp and frame number between 10 to 800 mAs. The field of view (FOV) size is adjustable between 50 mm (diameter) × 50 mm (length) to 442 mm × 235 mm using the offset imaging protocol. The voxel size of the device is 75 µm.

Five of the ten scanning protocols investigating the impact of kVp to the image quality were performed using standardised ED. The normalization of the ED was accomplished by adjusting the mAs for each kVp accordingly. The image quality resulting from “low ED” protocol (protocol 6) was acquired by using 90 kV and 100 mAs exposure parameters. The protocols 7–10 were attained by using the highest available mAs value for kVp range between 90 kVp, 100 kVp, 110 kVp and 120 kVp to investigate the impact of high ED to CNR and clinical image quality. The scanning protocols are presented in Table 2.

### Dose measurements and technical image quality assessment

The ED assessments were performed on an anthropomorphic phantom with cervical vertebrae RANDO SK150 phantom (Radiation Analogue Dosimetry System; The Phantom Laboratory, Salem, NY, USA). (Fig. 1B). The measurements were carried out according to a previous study [23] by using a mobile MOSFET device TN-RD-70-W20 comprising one TN-RD-38 wireless Bluetooth transceiver, four TN-RD-16 reader modules, twenty reinforced high-sensitivity TN-1002RD-H dosimeters and TH-RD-75 M software (Best medical, Ottawa, ON, Canada). Prior to the measurements, the MOSFET dosimeters were calibrated using a RADCAL 1015 dosimeter equipped with a RADCAL 10X5-6 ionization chamber (Radcal Corporation, Monrovia, CA, USA) according to previous studies by Koivisto et al. [23, 24].

Technical image quality indicators of each protocol were acquired according to a study by Ludlow et al. [25] using a QUART DVT_AP phantom (Fig. 1B) and QUART DVT_TEC (QUART GmbH, Zorneding, Germany). The modulation transfer function (MTF) is commonly used to assess the spatial response of imaging system. The MTF is defined as the absolute value of the system optical transfer function which at zero frequency is normalized to unity [26]. CNR is a measure of image quality based on contrast that can be formulated as follows:

$$CNR=\frac{\left|\mu F-\mu B\right|}{\sigma B}$$

where µF and µB are the mean foreground and background pixel values, respectively, and $\sigma$ B is the standard deviation of the background pixel values [27]. The QUART DVT_AP phantom consists of 16 cm diameter cylindrical slabs of Plexiglas with PVC and air elements configured to permit measurements of CNR and MTF 10% based on a standard DIN6868-161 [28]. Results were calculated from the measurements in a user guided, semi-automatic manner from DICOM slices selected from the volume. Three DICOM slices of each volume were measured, and the results were averaged. The QUART DVT_AP phantom is presented in Fig. 1C.

#### Figure of merit

In this study, the figure of merit (FOM) value was calculated to assess the diagnostic efficacy of the image quality versus the ED obtained using different imaging protocols. The FOM value was calculated using the following equation described by Ogden et al. [29]

$$FOM= \frac{{CNR}^{2}}{ED}$$

where ED is the effective dose.

### Clinical image quality assessment

All images acquired from five temporal bone specimens using 10 different imaging protocols were viewed in Planmeca Romexis Viewer (Planmeca Oy, Helsinki, Finland) and analyzed for eight anatomical landmarks: malleus head, incudomalleolar joint, incudostapedial joint complex (head of stapes and lenticular process), stapes superstructure (stapes anterior crus, stapes posterior crus and stapes footplate), facial canal and long process of incus. In order to attain quantitative evaluation of image quality, the aforementioned anatomical landmarks were evaluated by three evaluators (one otoradiologist, two otosurgeons) using a rating system from 5 to 1 in descending order similarly to a previous study by Zou et al. [30] as follows in Table 1. The anatomical landmarks were presented to the evaluators one bone and one protocol at a time in a randomized order. All the evaluators were blinded to each protocol, and they did their evaluation work independently.

**Table 1:** Rating system by Zou et al. (2015) used in this study

5	Very good delineation of structures and excellent quality
4	Clear delineation of structures and good image quality
3	Anatomic structures still fully assessable in all parts and acceptable image quality
2	Structures identified but no details assessable and results in insufficient image quality
1	Anatomic structures not identifiable due to poor image quality

### Reconstructing 3D objects from cone beam projections

A 3D volume reconstruction in CBCT differs from MSCT. In MSCT, reconstructed 3D volume is assembled from individual axial slices with a noted mathematical technique. With a flat 2D X-ray area detectors and cone beam shaped geometry, 3D volume in CBCT is reconstructed from 2D projection data [2] using Planmeca Romexis Viewer application.

### Statistics

Average rates and their standard deviations (SD) of all anatomical landmarks of all the five bones imaged with the 10 protocols were calculated. After that, a two-way analysis of variance (ANOVA) followed by Tukey’s multiple comparisons test (GraphPad Prism v8.0, San Diego, CA, USA) was conducted to compare the 10 imaging protocols among themselves.

## Results

### Technical image quality and effective dose

The exposure protocols, CNR, MTF, FOM and EDs of different protocols are presented in Table 2**.**

**Table 2:** Exposure protocols used for assessing subjective image quality, CNR, MTF and effective dose

Protocol nr	1	2	3	4	5	6	7	8	9	10
Tube voltage (kVp)	80	90	100	110	120	90	90	100	110	120
Q (mAs)	450	280	200	140	100	100	450	450	300	300
Tube curr. (mA)	32	40	40	40	32	36	36	32	28	22
Exp. time (s)	14.1	7.0	5.0	3.5	3.1	2.8	12.5	14.1	10.7	13.6
Voxel (µm)	75	75	75	75	75	75	75	75	75	75
Scan angle	210º	210º	210º	210º	210º	210º	210º	210º	210º	210º
Pulsed	yes	yes	yes	yes	yes	yes	yes	yes	yes	yes
Frame number	500	500	500	500	500	500	500	501	501	502
FOV height (mm)	50	50	50	50	50	50	50	50	50	50
FOV diameter (mm)	50	50	50	50	50	50	50	50	50	50
CNR	10.5	10.3	10.1	8.6	7.9	5.3	12.4	14.2	13.5	14.3
MTF	1.49	1.54	1.60	1.60	1.62	1.51	1.50	1.41	1.55	1.57
FOM	2.1	2.0	1.8	1.4	1.2	1.5	1.8	1.6	1.6	1.3
Effective dose (µSv)	52.8	53.8	56.0	52.7	52.7	19.2	86.5	125.9	112.9	158.0

CNR = contrast to noise ratio; MTF = modulation transfer function; Q = tube current exposure time product; FOV = field of view; FOM = figure of merit = CNR^2^/Effective dose

### Subjective image quality

**Fig. 2:**
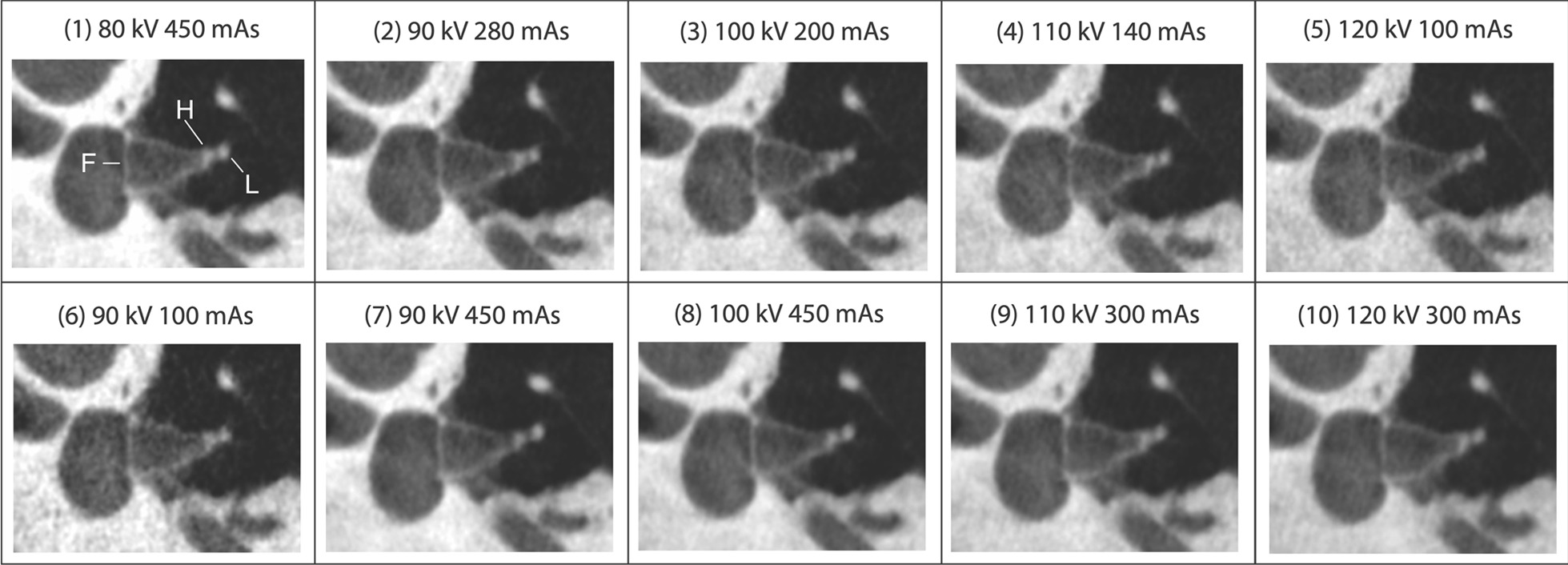
Stapes superstructure of one particular specimen imaged with the 10 different protocols. F, stapes footplate; H, head of stapes; L, lenticular process

In Fig. 2, stapes superstructure of one particular specimen is imaged with the 10 different protocols. By comparing the protocols’ ability to make the fine structures of stapes distinguishable, differences in protocol modalities’ imaging capacity can be demonstrated.

The average subjective mean image qualities and their SDs of three evaluators of all anatomical landmarks using all investigated imaging protocols are presented in Table 3, where seven-tiered colour coding is used to demonstrate the variability of the average subjective image qualities.

**Table 3:**
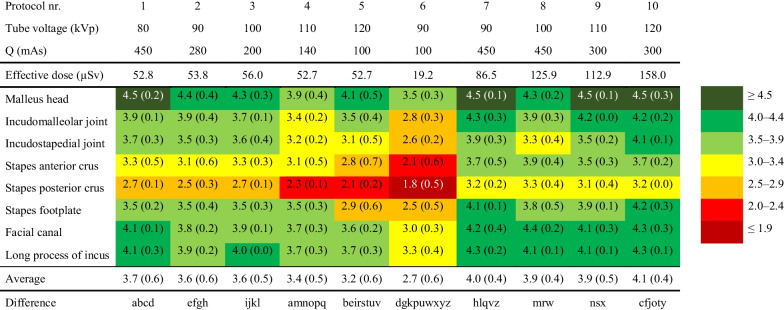
Clinical image quality demonstrated by average levels of three evaluators using a rating system from 5 to 1 in descending order.

Seven-tiered colour coding is used to demonstrate the variability of the average subjective image qualities. Data are mean (SD), *n* = 3. For the statistical significance of the difference, *p* < 0.05 (c, e), *p* < 0.01 (a, i, l), *p* < 0.001 (h, j), and *p* < 0.0001 (b, d, f, g, k, m, n, o, p, q, r, s, t, u, v, w, x, y, z).

A two-way ANOVA was conducted to compare the 10 imaging protocols. Statistical significances of the differences between used protocols are represented in the bottom row of the Table 3. SDs of the averages of the three evaluators varies between 0.0–0.7. It can be observed that the malleus head landmark has been evaluated the best quality structure (averages in all protocols between 3.5–4.5) getting the highest average (4.5 ± 0.2) of all of the landmarks in protocols 1, 7, 9 and 10.

Protocol 6 resulted in the lowest average subjective image quality 2.7 ± 0.6 of the ten protocols (*p* < 0.0001, when compared to protocols number 1, 2, 3, 4, 5, 7, 8, 9 and 10). This can be well explained by the lowest kVp (90 kVp) and lowest charge (100 mAs). On the other hand, protocol 10 results in the highest average of subjective image quality 4.1 ± 0.4 (*p* < 0.05, when compared to protocol number 1; *p* < 0.001, when compared to protocol number 3; *p* < 0.0001, when compared to protocols number 2, 4, 5, and 6). The good image quality results from using high ED and mAs values. When comparing the constant ED protocols from 1 to 5, it can be observed that the protocol 1 resulted in the highest average subjective image quality 3.7 ± 0.6 (*p* < 0.01, when compared to protocol number 4; *p* < 0.0001, when compared to protocols number 4 and 5). The differences in the mean subjective image quality between protocols 1 to 3 (80 kVp – 90 kVp – 100 kVp) is minimal. However, some organ specific image quality differences are seen for anterior crus and posterior crus of the stapes. Using a constant ED, the average image quality degrades when using tube voltages that are higher than 100 kVp. The protocols 4 and 5 offered a lower mean image quality resulting from using 110 kVp and 120 kVp. This phenomenon is seen especially for anterior crus and posterior crus of the stapes.

Notably, in protocols from 1 to 5 with constant ED, increase in mAs increases also average subjective image quality (*p* < 0.05 between protocols number 2 and 5; *p* < 0.01 between protocols number 1 and 4, 3 and 5; *p* < 0.0001 between protocols number 1 and 5) but the trend is opposite when kVp increases (*p* < 0.05 between protocols number 2 and 5; *p* < 0.01 between protocols number 1 and 4, 3 and 5; *p* < 0.0001 between protocols number 1 and 5).

Protocols between 7 to 10 offered comparable average subjective image quality (on average 3.9–4.1; no statistically significant differences) regardless of the large (86.5–158.0 µSv) ED variations. While the ED protocol 7 was about 45% smaller than in protocol 10 (158.0 µSv) there was no difference between average image quality of protocols 7 (4.0 ± 0.4) and 10 (4.1 ± 0.4). In addition, when comparing protocols 3 and 8 with EDs of 56.0 µSv and 112.9 µSv, respectively, the impact of 2.24-fold ED to the difference in the image quality between specific anatomical regions were moderate.

As a result, it can be concluded that with the exception of protocol 6 all other protocols demonstrated in Table 3 are competent to image fine structures in middle ear. However, with some of the protocols, the smallest structures, such as the footplate and the anterior crus and posterior crus of the stapes, can be distinguished most precisely. With a moderate ED, we can say that protocol number 7 has a good average image quality. Using the standardized ED, the protocols using 80 kVp to 100 kVp offered the best image quality. Furthermore, this kVp range offered the best image quality efficacy (FOM) i.e., the highest CNR^2^ versus the ED.

**Fig. 3:**
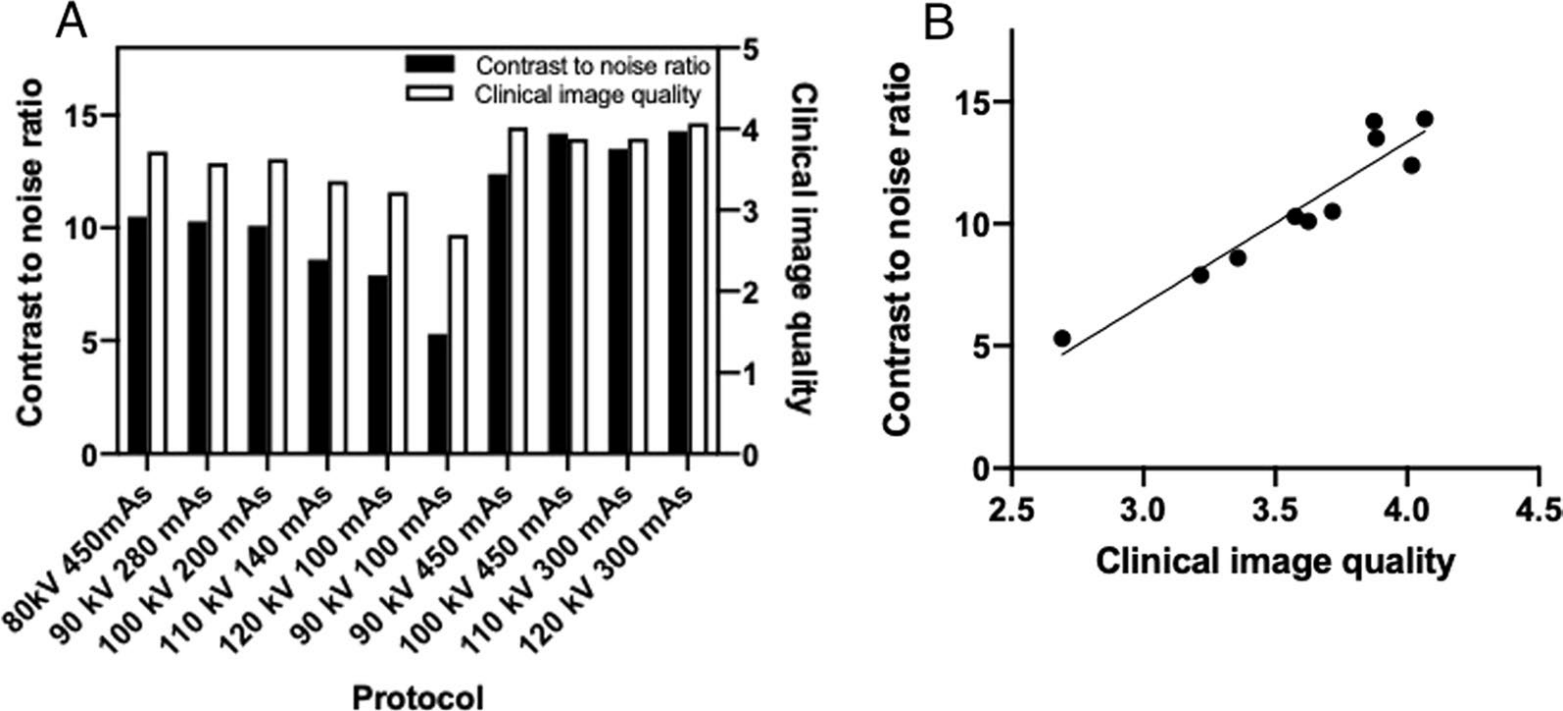
**A** The contrast to noise ratio and average clinical image quality of all anatomical landmarks using all investigated imaging protocols. **B** The correlation between contrast to noise ratio and average clinical image quality of all anatomical landmarks. Linear regression reveals significant correlation (R = 0.9059, F 76.98, *p* < 0.0001)

**Fig. 4:**
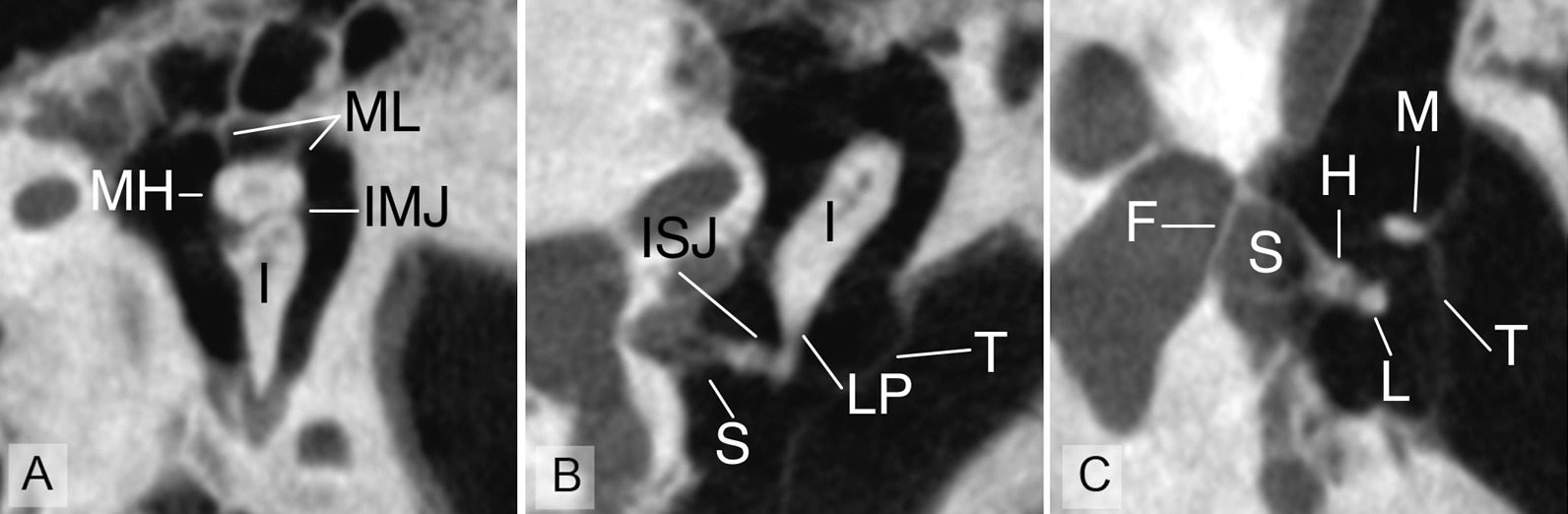
Anatomical landmarks used in this study imaged using protocol number 7 (90 kV 450 mAs). All three images are from same (but different than in Fig. 2) specimen. **A** I, incus; IMJ, incudomalleolar joint; MH, malleus head; ML, malleolar ligaments. **B** ISJ, incudostapedial joint; LP, long process of incus; S, stapes superstructure; T, tympanic membrane. **C** F, stapes footplate; H, head of stapes; L, lenticular process; M, malleus

In Fig. 3A, the CNR and average clinical image quality of all the anatomical landmarks using all investigated imaging protocols are plotted in the same figure. In linear regression model, a positive correlation (R = 0.9059, F 76.98, *p* < 0.0001) between CNR and average clinical image quality of the protocols was observed (Fig. 3B). Protocol 7 seems to have both a good average image quality and a competitive CNR value. As a compromise of average clinical imaging quality and an appropriate ED, the protocol 7 was chosen the best protocol used in this study. In Fig. 4, different anatomical landmarks of the middle ear are imaged using protocol 7.

### 3D reconstructions

**Fig. 5.**
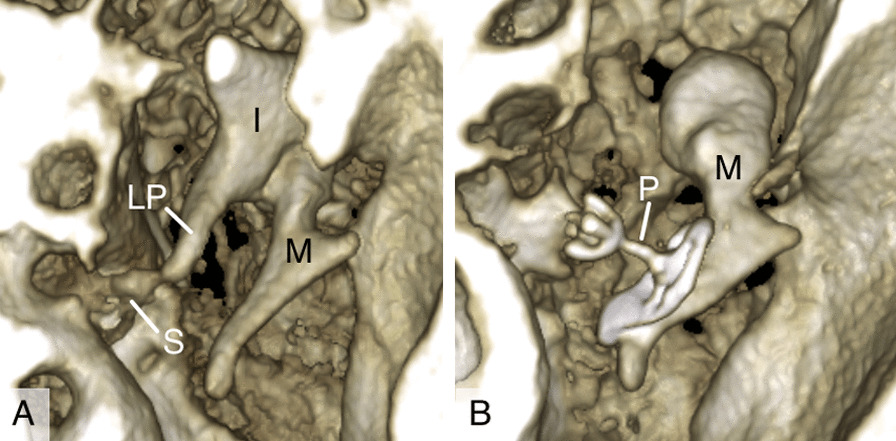
3D reconstructions of **A** stapes superstructure and **B** titanium partial ossicular chain prosthesis. I, incus; LP, long process of incus; M, malleus; P, partial ossicular replacement prosthesis; S, stapes superstructure

Figure 5 illustrates and example of 3D reconstructed ossicular chain imaged with protocol 7. As seen in Fig. 5A microanatomy of malleus and incus are well visible. Stapes superstructure may be distinguished (Fig. 5A) but the details are more visible in 2D images (Fig. 2). In addition, titanium partial ossicular chain replacement prosthesis is clearly imaged (Fig. 5B).

## Discussion

CBCT is a good option for imaging middle ear structures because of its good resolution capabilities and lesser EDs compared to MSCT [1, 6–9]. In this study, we have shown that the new Planmed XFI® full body CBCT device seems to be very suitable for middle ear imaging*.* No reconstruction parameters or image acquisition parameters other than the kVp and mAs were changed in the current study. A good correlation between CNR and subjective clinical image quality was discovered. The least CNR was achieved by combining the least kVp and mAs in protocol 6 with the least ED (19.2 µSv), too. The highest CNR among all these ten protocols studied resulted from using 120 kVp and 300 mAs in protocol 10 that yielded in the highest ED (158.0 µSv). Same CNR value can, on the other hand, be achieved by combining different kVp and mAs values as mentioned by Ogden et al. [29].

EDs of the constant dose protocols (protocols from 1 to 5) used in this study ranged between 52.7–56.0 µSv. In protocols from 6 to 10, EDs ranged from 19.2 µSv to 158.0 µSv. To compare, an ED of a full size panoramic radiograph is in all essentials 17.6 µSv [31] and middle ear MSCT is roughly between 1.99 and 2.33 mSv [32]. Measuring the EDs, other types of phantoms have been also used. A study by Ludlow et al. [25] compared ATOM type child and adult phantoms and found out child phantom EDs to be 36% greater when imaging with i-CAT FLX CBCT device.

The MTF varies quite a little (1.41–1.62) between all the ten protocols observed due to the same voxel size (75 µm) used for imaging. The aforementioned spatial resolution (voxel size) of this particular full body CBCT device is clearly better than in MSCT devices in current clinical use [11], which is a strength of this particular study.

During the last years, an increasing number of studies focusing on middle ear imaging using CBCT have been published. Many studies of inner ear and cochlear implant CBCT imaging have also been published recently but also a little earlier [8, 30, 33–35]. Comparing MSCT and CBCT, CBCT images have been evaluated better in image quality and visualization of fine bony structures with lower EDs [6, 7, 9]. A previous study by Dahmani-Causse et al. [9] brought up MSCT’s tendency to overestimate stapes footplate thickness. Previous studies have also introduced novel CBCT devices in middle ear imaging. With a cylindrical polymethyl methacrylate phantom, a study by Pauwels et al. [17] determined an optimal kVp setting at a fixed radiation dose and studied relationships between kVp, mAs and CNR. Concerning the lower image quality, they found out a dose reduction of mAs to be more efficient than an equivalent reduction of kVp. In a study by Zou et al. [22], human cadaver temporal bones were imaged with a new CBCT device with optimized imaging parameters and the anatomical landmarks in the images were measured. They found that particular CBCT device to be competent in imaging fine structures of the middle ear.

A clinically oriented study by Güldner et al. [36] evaluated CBCT’s visualization properties of different middle ear anatomical landmarks of 204 real human patients with chronic middle ear disease or conductive hearing loss and found out significant differences only in small bony structures when trying to detect pathological variations from anatomical structures. In that study, the CBCT images included middle ear soft tissues with their pathological variations. A study by Hodez et al. [1] also found out fine bony structures to be visualized very well but, on the other hand, also noted opacities in interpreting soft tissue variations due to image noise. Thus, it is important to find an imaging protocol that is sufficient regardless of soft tissue opacification in middle ear.

### Imaging of excised temporal bones instead of whole heads

This study was conducted using excised unfixed cadaver temporal bones and the EDs were assessment with a phantom lacking soft tissue structures and, therefore, we evaluated mainly interfaces between air and bone. In clinical context, soft tissue opacifications of middle ear can differ. An interpretation of the results should be carried out with care since the results were obtained using excised temporal bone and not the whole head. However, we have assessed the impact of soft tissue equivalent using head soft tissue equivalent water sack to the image quality for protocol 7 (90 kVp 450 mAs) and observed only minor losses of image quality. This phenomenon corresponds to the results of a study by Zou et al. [30] where they did not find significant changes in temporal bone CBCT image quality when additional water sack was used. When scanning these specimens, the position and spatial orientation of the bones can have been quite unnatural when reflecting it to a patient use.

The whole head causes beam hardening artifacts when using a typical CT scanner. However, the full body CBCT scanner of the current study has an added 2.5 mm Al + 0.5 mm Cu filtration resulting in a less pronounced beam hardening effect for whole head imaging than in typical CT device. However, due to the added filtrations the mean photon energy is higher than that e.g., for a GE Revolution CT scanner that uses total 4.2 mm Al equivalent filtration. The 2.5 mm Al + 0.5 mm Cu filtration of the CBCT device improves the radiation transmission and reduces the patient ED. Furthermore, this phenomenon reduces the differences in the image quality between temporal bone specimen and the whole head imaging.

### Clinical use of the device

In clinical use, the radiation dose should be as reasonable as possible. With this novel full body CBCT device we found imaging with protocol 7 (90 kV and 450 mAs) resulting in adequate CNR and subjective image quality with moderate ED. With the least ED and CNR of the protocols studied, protocol number 6 (90 kV and 100 mAs) could also offer a good clinical usefulness, for example, in postoperative imaging or in situations when a good resolution in stapes superstructure, especially the posterior crus, is not needed.

Patient movement is one of the most challenging causes for image quality degradation. In this preclinical study when scanning with the protocol 6, the exposure time was only 3 s which could be an advantage when scanning pediatric patients or other patients with a poor cooperation acquirement. The exposure times used in this study can also differ from patient use. Longer exposure time increases risks of artefacts caused by patient movement. The current full body CBCT scanner uses “Correction Algorithm for Latent Movement” (CALM) (Planmed Oy, Helsinki, Finland) which can analyse and compensate for patient movement in CBCT images. CALM restores the consistency of the X-ray measurements by tracking the patient movement resulting in a sharper final image. In the present study there was no need to use patient movement correction algorithm.

Nowadays, most of the CBCT devices are dedicated to dental, maxillofacial, paranasal sinus and otologic imaging. Importantly, the novel CBCT device studied here is designed for whole body imaging and in the current study, we found that it is suitable for middle ear imaging. In the future, it is possible that full body CBCT devices will become more common since they will enable otologic, maxillofacial, dental, and orthopaedic imaging with a single device, and it will be important to describe their performance in different organ systems including otologic imaging.

### 3D volume

To create 3D images, current MSCT devices and their image viewer software in clinical use need more experience and proficiency than creating 3D images from CBCT volume data. In our experience, the viewer software used in this study seems to be easy to use also for beginners. For example, the 3D volume is viewed automatically, and it can be manipulated using a simple computer mouse. Therefore, 3D volume of CBCT is more achievable to clinicians and not only for radiologic professionals. In the future, this feature could be easily utilized in preoperative planning, postoperative reviews, and 3D printed middle ear prosthesis designing.

## Conclusion

This study shows that the new Planmed XFI® full body CBCT device has potential in middle ear imaging. Based on the results of the current study, we are now able to estimate the best parameters for temporal bone imaging in the clinical setting. Detailed 2D images and 3D volumes can be obtained with moderate EDs and easy-to-use software. More studies in clinical context focusing on, for example, preoperative planning with CBCT should be conducted. Furthermore, the results of this study could be used to optimize the ED by selecting appropriate exposure parameters depending on the diagnostic task.

## Data Availability

The datasets used and/or analyzed during the current study are available from the corresponding author on reasonable request.

## Abbreviations

ANOVA: Analysis of variance
CBCT: Cone beam computed tomography
CNR: Contrast to noise ratio
CT: Computed tomography
ED: Effective dose
FOM: Figure of merit
FOV: Field of view
kVp: Tube voltage
MRI: Magnetic resonance imaging
MSCT: multi slice computed tomography
MTF: modulation transfer function
mAs: tube current exposure time product
Q: tube current exposure time product
SD: standard deviation
SNR: signal to noise ratio
